# Topical mycophenolate for the treatment of uveitis-associated inflammation

**DOI:** 10.1186/s12348-026-00569-y

**Published:** 2026-02-06

**Authors:** Jyoti Chauhan, Ermanno Gherardi, Hae Lin Jang, Shiladitya Sengupta

**Affiliations:** 1 Center for Engineered Therapeutics, Department of Medicine, Brigham and Women’s Hospital, Harvard Medical School, 65 Landsdowne Street, 317, Cambridge, MA 02139 USA; 2 Department of Molecular Medicine, University of Pavia, Pavia, Italy; 3 Vyome Therapeutics Inc., Cambridge, MA USA

## Abstract

**Objective:**

Uveitis refers to the inflammation of the uveal tract of the eye (iris, ciliary body and choroid). In the developed world, it accounts for 10–15% of all cases of blindness. Anterior uveitis accounts is the most common form of uveitis. There is an unmet need for a topically administered non-steroidal drug to treat anterior uveitis.

**Methods:**

We tested two topical formulations of mycophenolate (MPA), an inhibitor of inosine monophosphate dehydrogenase (IMPDH) enzyme, as a potential steroid-sparing treatment for uveitis. We studied first the binding of MPA to a three-dimensional model of human IMPDH2 generated with AlphaFold 3. Next, we formulated mycophenolate sodium as an aqueous suspension and mycophenolate mofetil as an ointment. Permeability of mycophenolate through the corneal barrier was measured using a Franz cell assay using a goat eye cornea as the membrane. Drug concentration in the different compartments of the eye involved in anterior uveitis was measured using liquid chromatography-tandem mass spectrometry (LC-MS/MS). Both formulations were tested for acute ocular irritation in vivo, and efficacy in a rabbit model of uveitis.

**Results:**

The AlphaFold 3 model of IMPDH2 offered a detailed map of the MPA binding site. MPA makes hydrogen bonds to main chain atoms of S276 and G326 and side chain atoms of S276, T333 and Q441 as well as hydrophobic interactions with S276, G415 and Y430. The computational analysis shed new insights on the mechanism of mycophenolate inhibition and allosteric regulation of the enzyme. Mycophenolate was stable over 6 months in both suspension and ointment. Topical application of mycophenolate sodium 1% and 2% suspension eye drop exhibited a drug flux of 81·81ug/cm^2^ and 140.42 ug/cm^2^, respectively, through the corneal barrier, greater than 11.01 ug/cm^2^ and 26.54 ug/cm^2^ achieved with mycophenolate mofetil 1% and 2% ointments, respectively. The formulations were non-irritant to eyes of New Zealand white rabbits. No systemic clinical signs of toxicity and necropsy findings were observed. Mycophenolate sodium 2% suspension-treated group showed significant reduction (*p* < 0.0010) in the uveitis score with reduced leukocyte counts in the anterior chamber compared to vehicle control and was not statistically different from positive control prednisone steroid (*p* = 0.44).

**Conclusion:**

Topical mycophenolate sodium 2% suspension could emerge as an effective non-steroidal treatment for anterior uveitis and merits clinical evaluation.

**Supplementary Information:**

The online version contains supplementary material available at 10.1186/s12348-026-00569-y.

## Introduction

Uveitis is a devastating ophthalmic disease, characterized by the inflammation of the uveal tract (iris, ciliary body and choroid) of the eye. It is a major cause of blindness in both developed and developing countries [1, 2], and is implicated in causing 30,000 new cases of legal blindness, annually, in United States alone [3]. The worldwide prevalence of uveitis is estimated to be 115–204 per 100,000 individuals [4]. Based on the anatomical site of inflammation, uveitis can be classified as (i) anterior (inflammation of the ciliary body and iris), (ii) intermediate (vitreous inflammation), (iii) posterior (inflammation of the retina and choroid), and (iv) panuveitis, which includes all the above anatomies. Anterior uveitis is the most common type of uveitis seen in both adult and pediatric patients, accounting for up to 90% of cases in primary care and 50–60% of cases in tertiary care [5], with an estimated total of 241,665 cases in the US alone [6].

Approximately 50% of cases of uveitis are idiopathic in nature, i.e. with no clear underlying cause, while the rest can occur due to trauma, surgery, systemic autoimmune disorders such as Behcet’s disease, rheumatic diseases (Juvenile Idiopathic Arthritis, HLA-B27-associated), infectious etiology (toxoplasma, virus, treponema, etc), or certain medications such as TNF inhibitors and immune checkpoint inhibitors [7]. Corticosteroids (combined with antimicrobials for infectious uveitis) are the mainstay of therapy in uveitis for their broad anti-inflammatory role. They are administered either topically for anterior uveitis or systemically (via oral, periocular or intravitreal injections and sustained-release implants) for anterior uveitis not responding to topical applications, intermediate and posterior uveitis. However, the long-term use of corticosteroids is limited by elevation of intraocular pressure, risk of secondary glaucoma, and cataract formation [8], which is particularly high risk in children because of the added risk of developing amblyopia secondary to cataracts. There is growing evidence that instilling topical glucocorticoids more frequently than twice a day substantially increases the risk of intraocular pressure elevation, potentially leading to glaucoma and subsequent visual field loss. This poses a significant challenge as uveitis has a high rate of relapse, i.e. ~39% relapse within 1.5 years after remission [3], necessitating repeated and prolonged treatments. Additionally, uveitic glaucoma is a common complication affecting some 20% of uveitis patients, which further complicates the use of corticosteroids in such patients.

In clinical practice, a wide range of systemic immunomodulatory drugs have been used to treat patients refractory to steroids. Adalimumab, a tumor necrosis factor-α-binding antibody, is FDA approved for the treatment of noninfectious intermediate uveitis, posterior uveitis, and panuveitis, and is administered subcutaneously [9]. Repository corticotropin injection (RCI) has been suggested to exert immunomodulatory and anti-inflammatory effects, and is FDA approved to treat ocular inflammation, including uveitis [10]. Similarly, methotrexate and mycophenolate are used off-label to treat steroid-refractory cases [11]. However, these therapeutics are administered systemically, and there is an unmet need for testing a topically administered immunomodulatory agent to treat anterior uveitis.

To address this need, we developed and tested two topical formulations of mycophenolate, which is currently approved by the FDA for prophylaxis of organ rejection [12]. The immune-privileged state of eye [13] is broken in uveitis and an inflammatory process develops characterized by intraocular infiltration of leukocytes [14]. Single cell RNA sequencing and immunohistology have shown infiltration of both T and B cells in aqueous and vitreous humor and in the uveal tract of patients [15] and the presence of ectopic lymphoid structures that further amplify the immune response [16]. Mycophenolate inhibits inosine monophosphate dehydrogenase (IMPDH) enzyme, which catalyzes the nicotinamide adenine dinucleotide (NAD)-dependent oxidation of inosine monophosphate (IMP) to xanthosine monophosphate (XMP), a key step in guanine nucleotide synthesis [12]. B and T cells depend on IMPDH activity to generate the guanosine nucleotide pool needed to initiate a proliferative response to mitogens or antigens. As a result, mycophenolate depletes both T and B cells [12]. Indeed, systemic off-label oral administration of mycophenolate has been shown to be effective in the management of uveitis [8]. However, systemic mycophenolate is frequently associated with major side effects (teratogenicity, gastrointestinal distress, neutropenia, lymphocytopenia, hepatitis and increased probability of opportunistic infections) [8] while only a fraction of the administered dose reaches the eye. We rationalized that a topical eye formulation could achieve the necessary concentration of mycophenolate in the ocular tissues for the treatment of anterior uveitis. Here we formulated mycophenolate into an ointment and an eyedrop and tested its efficacy in an in vivo model of uveitis.

## Methods

### Formulating topical mycophenolate

Mycophenolate mofetil 1 and 2% ointments were formulated using a mixture of light liquid paraffin (Drakeol 19), white soft paraffin (Merkur 500), lanolin and lanolin alcohol as ointment base (Table 1). Briefly, in step 1, precisely weighed quantity of Merkur 500, lanolin and lanolin alcohol were mixed and constantly stirred over a water bath maintained at 60–65°С. In step 2, mycophenolate mofetil (Obtained from Biocon ltd) was added to a part of Drakeol 19 under high shear homogenization (10,000 rpm) and was allowed to form slurry for 30 min. In step 3, the material of step 1 was allowed to cool down with stirring up to 50°С, and in Step 4, the slurry prepared in step 2 was added to molten phase of step 3 under stirring at 150 rpm. The remaining part of Drakeol 19 was used to rinse the high shear homogenizer and added to step 4 phase. Formulation obtained in step 4 was allowed to congeal at room temperature with stirring. We used mycophenolate sodium to prepare the 1 and 2% suspension eye drop. Glycerol, disodium edetate, polysorbate 80 and boric acid were first dissolved in purified water followed by addition of Carbopol 974P and mixing for 45 min. Mycophenolate sodium (obtained from Biocon) was dispersed in the phase obtained in the above step, mixing using high shear homogenizer at 10,000 rpm. Final weight makeup was done using purified water. Prednisolone acetate 1% suspension (Allergan) was used as the positive control. Bovine serum albumin (BSA) and complete Freund’s adjuvant were obtained from Sigma Aldrich. Mycophenolate sodium was obtained from Biocon (Bangalore), and mycophenolate mofetil was obtained from Clearsynth labs. Formulations were prepared in Vyome R&D laboratory.

**Table 1:** Composition and properties of mycophenolate formulations for topical ophthalmic application

**Mycophenolate mofetil ointment composition in Percentage (% w/w)**			
1%			2%
Mycophenolate Mofetil API		1.0	2.0
Light liquid paraffin (Ointment base)		27.3	26.3 (Drakeol 16)
White soft paraffin (Ointment base)		63.7	63.7 (Merkur 500)
Lanolin (Ointment base)		3.0	3.0
Lanolin Alcohol (Ointment base)		5.0	5.0
Description: Viscosity:			Off white ointment 6060.77 mPa.s
**Mycophenolate sodium (sod) suspension formulation composition in percentage (w/v)**			
		**1.0%**	**2.0%**
*Ingredient*	*Function*		
Mycophenolate sod.	API	1.00	2.00 acid
Polysorbate 80	Surfactant	0.25	0.25
Carbopol 974P	Thickening	0.30	0.30
Glycerol	Tonicity	0.586	0.40
Di-sodium EDTA	Chelating	0.10	0.10 EDTA
Boric acid	Preservative	1.00	1.00
Purified water	q.s. up to 100 ml		
Description:	White aq. suspension		
pH		6.62	6.87
Specific. Gravity		ND	1.017
Viscosity			181mPa.s

### Molecular docking studies

The sequences of human and hamster IMPDH2 (P12268, IMDH2_HUMAN and P12269, IMDH2_CRIGR), and trichomonad IMPDH (P50097, IMDH_TRIFO) were retrieved from the Uniprot database [17] and aligned using the fast Fourier transform procedure of Katoh et al. [18]. Docking of MPA onto an AlphaFold model of human IMPDH2 (AF-P12268–F1-model_v4) was carried out using the Boltz-1 implementation [19] of AlphaFold 3 [20]. Ligand-protein interactions were analysed with PoseEdit [21]. Tetrameric assemblies of human IMPDH2 were generated with GalaxyHomomer [22] and images of human IMPDH2 in complex with IMP and MPA were generated with Pymol [23].

### Corneal permeability assay

Cornea was gently dissected from fresh goat eyes obtained from abattoir. The cornea was sandwiched between the donor and receptor compartments in a Franz cell in phosphate buffer saline (pH 7.4). Each formulation was applied at a dose equivalent to 500 µg of active drug for 1% formulations and 1000 µg of active drug for 2% formulation. The drug flux through the corneal barrier was measured for 2 hours. The bioanalytical method to quantify mycophenolate mofetil and sodium was developed at department of drug metabolism, pharmacokinetics and clinical pharmacology, Eurofins Advinus Limited (report: n4892). Liquid chromatography-tandem mass spectrometry (LC-MS/MS) has gained prominence as a pivotal tool in therapeutic drug monitoring of mycophenolate [24]. Briefly, 1 g of eye tissue was weighed and mixed 5 parts (5 mL) of [1:5] 5% collagenase in phosphate buffer. This mixture was allowed to remain at room temperature overnight for homogenization. This homogenized matrix was used for further processing. Calibration curve standards and quality control samples were prepared by adding 2.5 μL of spiking solution into 47.5 μL of blank eye tissue homogenate and vortexed. The standard blank and standard zero samples were prepared by spiking 2.5 μL of diluent solution into 47.5 μL of interference-free blank plasma and vortexed. Internal standard (20 μL) was then added to all tubes except for standard blank sample (added 20 μL of diluent). Acetonitrile (1000 μL) was next added to the samples and vortexed for about 10 seconds, and centrifuged for 10 minutes at 14000 rpm at 4 °C. 10 μL of the supernatant was injected into the API 4000 LC-MS/MS system, in isocratic binary mode with Mobile phase A (5 mM ammonium acetate with 0.1% formic acid, v/v) and mobile phase B (Acetonitrile with 0.1% formic acid, v/v) in a 15% and 85% mix, and using a Kromasil C8 column. Test tissue was also similarly processed and compared against standard. Acceptance criteria for standard curve was that 75% of standards must be within ±15% (85 to 115%) of nominal concentration except for LLOQ, which should be within ±20% (80 to 120%) of nominal concentration. The method was found suitable for use within the range of 0.263 to 262 ng/mL for mycophenolate mofetil and 2.58 ng/mL to 2560 ng/mL for mycophenolic acid (Suppl. Fig. S1).

### Ex-vivo ocular pharmacokinetic studies using whole goat eye

To measure mycophenolate in different parts of the anterior eye, we performed the Franz cell assay using whole goat eye. Whole goat eye (from abattoir) was placed over Franz cell in PBS infused at 10 µl/min using microinfusion pump. A circular ring of 8.5 mm was placed over the cornea and the drug was applied inside the ring. After 7 min, the formulation was wicked away, and drug permeation through cornea was measured after extracting the perfused aqueous humor at different time points using a 23 G needle (Fig. 4a). At the end of the study (5 h post application), we isolated different tissues from the anterior eye and analyzed the total amount of drug in the tissue using the above bioanalytical method.

### Evaluation of eye irritation potential in vivo

The eye irritation/corrosion potential of mycophenolate mofetil topical ointment (1% w/w and 2% w/w) and mycophenolate sodium topical suspension (1% w/v and 2% w/v) was tested in New Zealand White rabbits (weight range 2.1817 to 3.4080 kg) in accordance with the OECD Guideline for Testing of Chemicals, Sect. 4, Health Effects, Number 405. “Acute Eye Irritation/Corrosion” 2017. Animal studies were performed at department of drug safety assessment, Eurofins Advinus Limited, under protocol (#004-July-2019) approved by the Institutional Animal Ethic Committee of Eurofins Advinus Limited in compliance with CPCSEA India. Both eyes of New Zealand White rabbits were examined one day prior to instillation of formulations with an ophthalmoscope (Welch Allyn). On test day, approximately one hour prior to instillation of the test item, a systemic analgesic agent (buprenorphine hydrochloride injection I.P.-0.3 mg/mL) was administered by subcutaneous injection, and approximately 5 min prior to instillation of the test item, 2 drops of topical anesthetic agent (proparacaine HCI 0.5%) were instilled in both the test and control eye. The formulations were applied (0.1 g or 0.1 mL) into the conjunctival sac of the left eye. The lids were held together gently for about one second to prevent any drug loss. The right eye of each animal was treated similarly with respective vehicle formulations to serve as the vehicle-treated control. After 24 h contact period, the treated eyes were irrigated with normal saline to remove the residual test item. Clinical signs of toxicity and pre-terminal deaths were observed four times (at hourly intervals) on the day of test item instillation and once daily on days 2 to 5 post-instillation. The eyes of each rabbit were examined at 1, 24, 48 and 72 h, post-instillation and the reactions were recorded. The eyes of each rabbit were examined using an ophthalmoscope and scored. In addition, all the treated eyes were examined using ophthalmic fluorescein sodium at 24 h post-instillation for all the rabbits. The grades of ocular lesions were assessed and scored on standard scales (Suppl. Table 1).

### Rabbit uveitis model

Male New Zealand white rabbits ( > 2 kg) were housed in under controlled environmental conditions with 12 h light and dark cycles and 20 ± 3 °C and 30–70% humidity. All the animals, other than the ones in the normal control group, were sensitized with BSA in complete Freund’s adjuvant emulsion at a dose of 1 mg/animal, administered subcutaneously at 3–4 sites on the back of rabbit on days 1, 3, and 5. After 9 days, all animals except normal control animals were rechallenged with intravitreal eye injection of 50 µg BSA in 100 µL of sterile water. The rabbits were observed for any inflammation for a period up to 3 h post intravitreal injection. The animals were randomized after 3 h of observation confirming uveitis in one eye (in contralateral eye, no convincing signs of uveitis were clinically demonstrated) into 4 different treatment groups with approximately equal grading score. The animals were treated with mycophenolate mofetil topical ointment (2.0% w/w, 100 mg), mycophenolate sodium topical suspension (2.0% w/v, 100 µl), prednisone suspension (1% w/v, 100 µl), or vehicle formulation (vehicle of mycophenolate sodium suspension, 100 µl) instilled topically twice daily with an interval of 6 h between two doses into one eye (which developed uveitis) from day 9 to day 13. The rabbits were monitored and scored for the induction of flare and accumulation of inflammatory cells, causing opacity of cornea. The scoring of eye under slit lamp was recorded at 0 h (before intravitreal injection), 3 h, 24 h, 48 h, 72 h and 96 h post intravitreal injection of BSA. On day 13, animals were treated with respective treatment group and were euthanized post ~6 h of last dose with excessive anesthesia. The following parameters were monitored: (i) uveitis scoring using slit lamp; (ii) representative eye photographs from each treatment group; (iii) white blood cell and differential leukocyte count in aqueous humor at terminal using an autoanalyzer (*n* = 3/group at the end of study period) and histopathology of eye (*n* = 3/group at the end of study period). Animal studies were performed at department of drug safety assessment, Eurofins Advinus Limited, under protocol (#EAL-185-Uveitis_RB058/Jan2020) approved by the Institutional Animal Ethic Committee of Eurofins Advinus Limited in compliance with CPCSEA India.

### Statistical analysis

All statistical analysis was performed using Graphpad Prizm version 10. Data are shown as mean ± SE with at least 3 independent replicates. ANOVA followed by Tuckey’s post-Hoc test was used to test for significance between groups. A threshold of p less than alpha of 0.05 was considered as statistically significant.

## Results

### Binding of mycophenolate to human IMPDH2

The major isoforms of IMPDH1 and IMPDH2, encoded by two separate genes [25] share 84.05% sequence identity and display a similar, overall two-domain structure consisting of: (i) a large catalytic domain, (ii) a flexible linker connecting the catalytic and a regulatory domain and, (iii) a smaller regulatory domain composed of two copies of a ⍺/β/⍺ module (CBS) homologous to the one found in cystathionine beta-synthase. IMPDH2 is upregulated in proliferating cells, such as activated lymphocytes, while IMPDH1 plays a housekeeping role. Mycophenolate preferentially inhibits IMPDH2 [26], giving it selectivity for treating conditions driven by an overactive immune system.

**Fig. 1:**
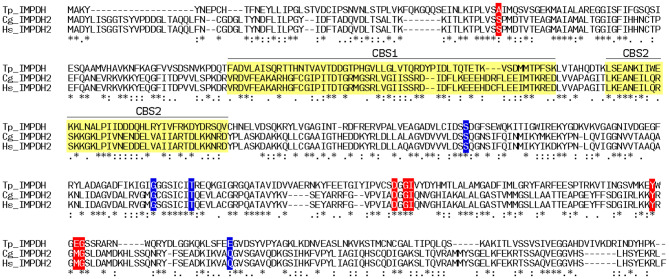
Alignment of the protein sequences of *T phoetus* IMPDH, *Cgriseus* IMPDH2 and human IMPDH2. The sequences of the two CBS motifs that constitute the regulatory domain of the enzyme are shown as CB1 and CB2 boxes. Key residues involved in binding IMP or MPA are shown on red or blue background. Human IMPHD2 shares 35.71 and 98.83% sequence identity with *T phoetus *IMPDH and *Cgriseus *IMPDH2, respectively. The sequences used for the alignment were P12268 (IMDH2_HUMAN), P12269 (IMDH2_CRIGR) and P50097 (IMDH_TRIFO) from uniprot (14) and alignment was constructed using the fast fourier transform procedure of Katoh et al. [15]

Although MPA is clinically used, the binding site of MPA in human IMPDH2, has not been fully characterized. Co-crystal structures of IMP and MPA in complex with the IMPDH enzymes of the unicellular parasite Tritrichomonas (*T phoetus)* [27] and Chinese hamster (*C griseus*) have defined the key residues involved in substrate and inhibitor binding. An alignment of the amino acid sequences of *T phoetus* IMPDH, *C griseus* IMPDH2 and human IMPDH2 is shown in Fig 1a in which the sequences of the two CBS modules are highlighted and the amino acids making hydrogen bonds to the IMP substrate or the MPA inhibitor are shown on red or blue background respectively. The alignment demonstrates that the residues responsible for polar interactions with the IMP or MPA are fully conserved in the hamster and human sequences (Fig. 1b).

**Fig. 2:**
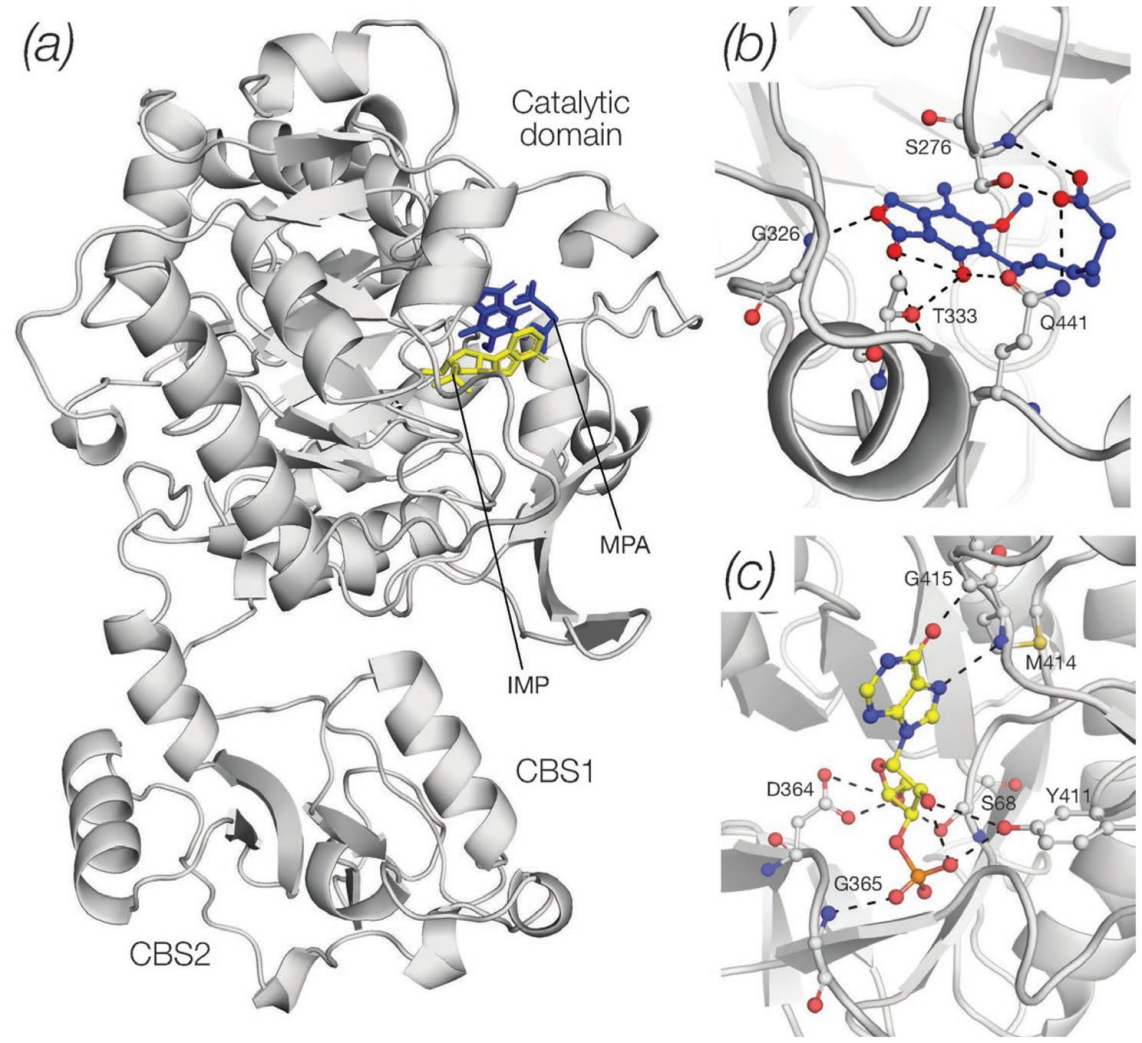
A 3D model of human IMPDH2 in complex with with IMP and MPA. (**a**) Overall structure showing the large catalytic domain of the enzyme and the regulatory domain extruded from the catalytic one. The latter is composed by two copies of the CBS module. The IMP and MPA ligands were bound to an AlphaFold model of human IMPDH2 (AF-P12268–F1-model_v4) using the Boltz-1 implementation of AlphaFold 3. (**b**) The hydrogen bond network of MPA. The inhibitor occupies the nicotinamide subsite of the NAD+ binding site and is shown at the centre of the image (carbon atoms: blue, oxygen atoms: red). (**c**) The hydrogen bond network of IMP (carbon atoms: yellow, nitrogen atoms: blue, oxygen atoms: red). Ligand-protein interactions were analysed with PoseEdit and images were generated with Pymol

We next used a full length AlphaFold model of human IMPDH2 [28] and the novel, diffusion-based architecture of AlphaFold 3 (AF3) [20], in order to model the binding sites of IMP and MPA in human IMPDH2 at an atomic level. The overall structure of the human IMPDH2-IMP/MPA complex is shown in Fig. 2a (the IMP molecule is shown in yellow, MPA in blue, the two CBS modules of the regulatory domain are indicated). Figure 2b and c show details of the MPA and IMP binding sites respectively. Binding of MPA to human IMPDH2 involves a hydrogen bond network with S276, G326, T333 and Q441 (Fig. 2b) and further hydrophobic contacts with S276, G415 and Y430. Thus, as seen earlier in the crystal structures of MPA in complex with the Tritrichomonas (*T phoetus*) [27] and hamster (*C griseus*) [26] enzymes, MPA occupies the nicotinamide subsite of the NAD+ substrate.

**Fig. 3:**
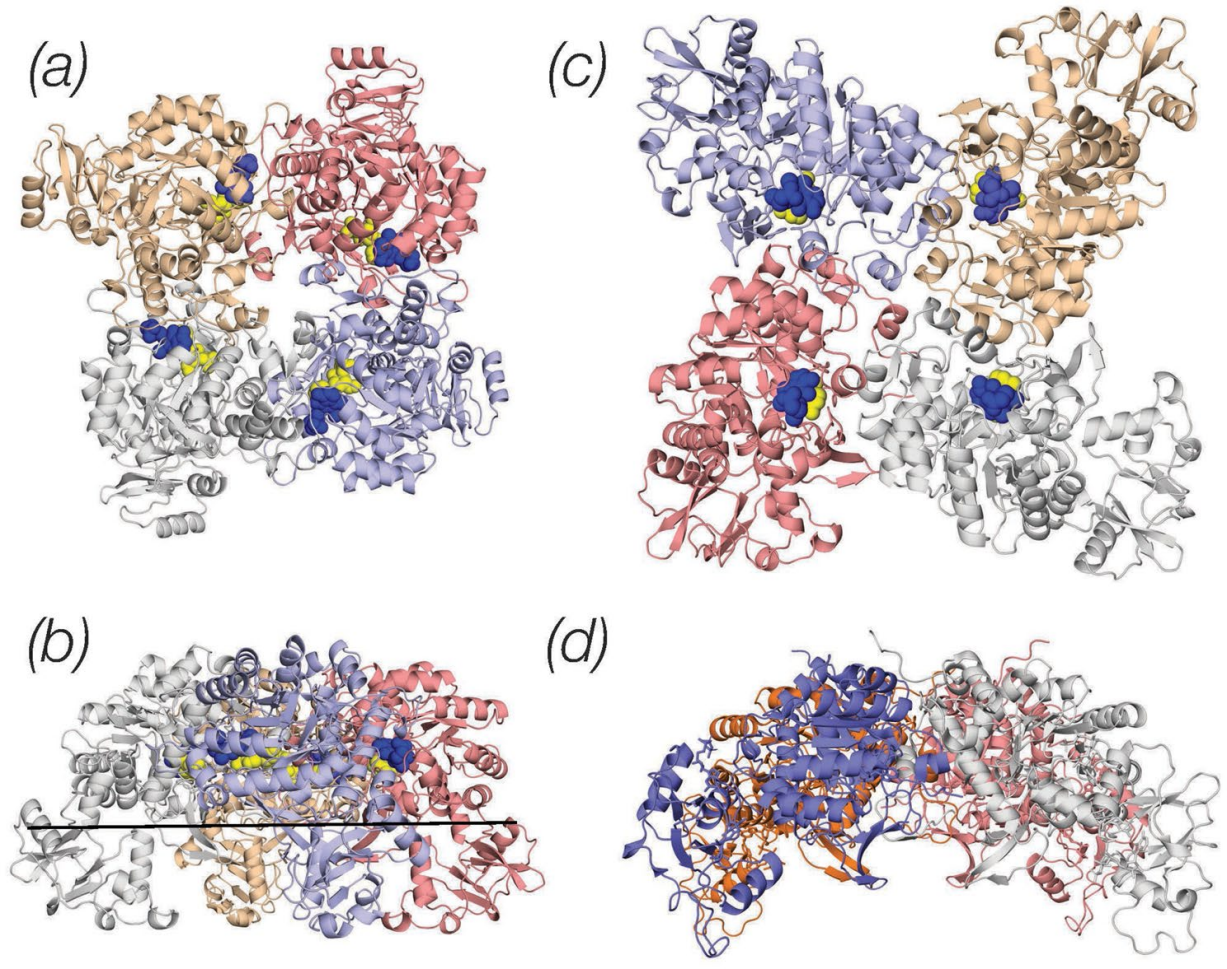
Tetrameric assemblies of human IMPDH2. The atomic coordinates of the IMPDH2 monomer shown in Fig. 2 was used in order to build a tetrameric assembly of the holo-enzyme. The position of the MPA and IMP ligands (shown as blue and yellow spheres respectively) illustrates that the catalytic pocket of the IMPDH2 enzyme is located in close proximity of the inter-protomeric interfaces. Two models of the tetrameric assembly are shown: the first one (panels **a**/**b**) is energetically favoured and displays the regulatory domains at a sharp angle relative to the catalytic domains. As a result the holoenzyme assumes the staple structure (shown in panel **b**). In the second model shown in panels **c**/**d**, energetically unfavored, the regulatory domains lie nearly horizontally relative to the catalytic domains and as a result the holoenzyme acquires a more open conformation. Tetrameric assemblies of human IMPDH2 were generated with GalaxyHomomer and images were generated with Pymol

Early crystallographic studies had clearly established that IMPDH enzymes assemble in stable tetramers in which the catalytic domains of each protomer provide the building blocks and inter-protomeric interfaces [26, 27]. We modeled the tetrameric assembly of human IMPDH2 and confirmed that the human enzyme adopts a quaternary architecture similar to the one seen in the crystal structures [26, 27] in which the catalytic sites of the enzyme are arranged in close proximity of the inter-protomeric interfaces (Fig. 3; MPA inhibitor is shown as blue spheres, the IMP substrate as yellow spheres). In the model of the human IMPDH2 tetramer energetically most favored (Fig. 3a and b) the four regulatory domains are placed at a sharp angle relative to the catalytic domains and, as a result, the holoenzyme adopts a compact, stapled conformation (Fig. 3b). The flexible linker connecting the catalytic and regulatory domains, however, allow for more open conformations, such as the one shown in Fig. 3c that, although energetically less favored, are not disallowed and may reflect different functional states of the holoenzyme.

## Mycophenolate sodium suspension eyedrop exhibits higher corneal flux

Our theoretical analysis of human IMPDH2-ligand complexes based on the AlphaFold 3 architecture, complements and validates the experimental and clinical observations that mycophenolate could indeed regulate the immune response via IMPDH2 inhibition in humans. We next tested if mycophenolate applied topically on the eye could penetrate the corneal barrier. Although eye drops are preferred over ointments in the treatment of uveitis, we developed both formulations for testing. We formulated mycophenolate mofetil in an ointment and mycophenolate sodium as an eye drop (Table 1). The drug was found to be stable in both formulations over 6 months (Suppl. Table 2). To test the permeation through the corneal layer, we used a Franz assay using an isolated goat cornea as a model system. The formulations were applied on the top of the cornea, and the amount of drug in the receptor chamber of the Franz cell was measured, and drug flux was calculated as the total drug that permeated per square cm of area of drug application. As shown in Table 2, both formulations resulted in a concentration-dependent increase in mycophenolate levels in the receptor chamber. We did observe the conversion of mycophenolate mofetil to mycophenolic acid when applied on the corneal layer, consistent with the presence of carboxylesterases in ocular tissue [29]. Mycophenolate sodium eye drop exhibited greater drug flux through the cornea as opposed to mycophenolate ointment, resulting in significantly higher drug concentration in the receptor chamber.

## Ocular pharmacokinetics with topical formulations of mycophenolate

**Fig. 4:**
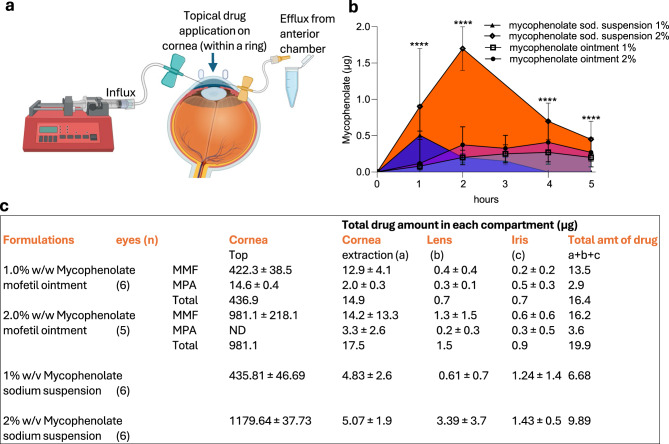
Ocular pharmacokinetics of mycophenolate in anterior eye. (**a**) Schematic shows the ex vivo preparation. Whole eye preparation was mounted in a Franz cell. The anterior chamber of the eye was cannulated with 30 G needles, one to infuse buffer and the other to withdraw sample from the anterior chamber. A circular ring (8.5 mm diameter) was placed over the cornea to retain the formulation. After 7 min, formulation that was not absorbed was wicked using cotton (and served as cornea top measurement). Schematic generated using Biorender. (**b**) Graph shows the concentration of mycophenolate (μg/ml) achieved in the anterior chamber at different time points after application of different formulations at doses equivalent to 500 μg of active drug for 1% formulation and 1000 μg of active drug for 2% formulation. Data shown are mean ± SD (*n* = 5–6). (**c**) Table shows the concentration of mycophenolate (μg/ml) in different anatomies of the anterior eye over 5 hours post-application. Data shown are mean ± SD (*n* = 5–6). ****p < 0.0001 vs 2% ointment using two-way ANOVA followed by Tukey’s multiple comparison test. MMF: mycophenolate mofetil; MPA: mycophenolic acid

We next validated the above observations using an ex vivo eye preparation, measuring the drug penetration into the anterior chamber of the eye following topical application. Whole goat eyes were placed in a Franz cell, and the formulations were applied topically. The drug influx was monitored by extracting aqueous humor from the anterior chamber (Fig. 4a). Consistent with the results from the corneal permeability assay, the application of mycophenolate sodium 2% suspension eye drops resulted in a higher drug concentration in the aqueous humor than with 2% ointment. Maximal concentration (Cmax) was reached earlier (between 1-2 h post application) and with a T_1/2_ between 2-3 h with the mycophenolate sodium 2% suspension eye drops as compared with the mycophenolate mofetil ointment, where Cmax was achieved between 3-4 h (Fig. 4b). We additionally measured drug levels in different anatomies in the anterior eye. As shown in Fig. 4c, the concentration in the iris, an anatomy that is impacted in uveitis, was statistically similar with all the formulations. We observed higher concentration of the drug in the cornea with the ointment, but this is likely due to the increased flux seen with the suspension eye drop vs ointments. Indeed, steroid suspension eye drops have been preferred over ointments for such reasons. The high drug concentrations retained on corneal surface also highlights the challenges of topical ocular delivery, especially in crossing the corneal barrier.

## Ocular efficacy and safety of mycophenolate topical formulations in vivo

We first tested the mycophenolate suspension and ointment 1 and 2% formulations for any potential to cause eye irritation using a rabbit eye model. There were no clinical signs of toxicity and pre-terminal deaths and no abnormality was detected at necropsy in any of the animals. There were no eye reactions observed in any of the tested formulations and vehicle formulations.

**Fig. 5:**
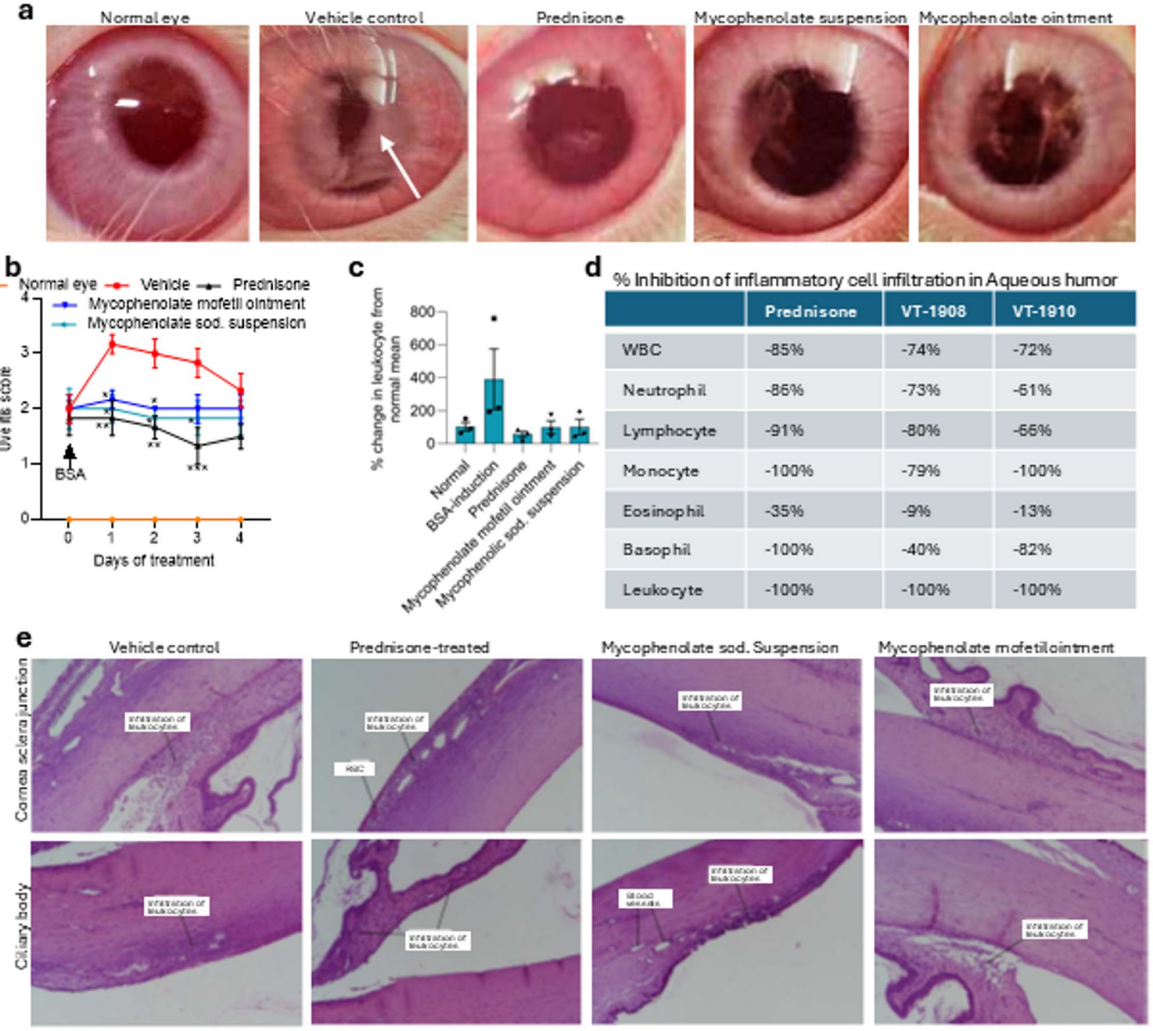
Topical mycophenolate is efficacious in treating uveitis in vivo. (**a**) Representative normal and uveitis-induced rabbit eyes treated with vehicle (for mycophenolate suspension eye drop) showing reduced opacity and flare consistent with immune cell infiltration (white arrow). Treatment with prednisone, mycophenolate sodium 2% suspension or mycophenolate mofetil ointment reduced the uveitic flare and corneal opacity. (**b**) Graph shows the uveitic score. Treatment with mycophenolate sodium suspension or mycophenolate mofetil ointment significantly reduced the uveitic score comparable to prednisone. Data shown are mean ± SD (*n* = 5–6, *p < 0.05, ***p < 0.001 vs vehicle-treated controls. ANOVA followed by Tukey’s test.) (**c**) Graph shows the mycophenolate and prednisone treatment-induced reduction in infiltrated leukocytes in the anterior chamber post BSA challenge in the eye in sensitized rabbits. Data shown are mean ± SD (*n* = 3). (**d**) Table shows the percentage reduction in different immune cells in anterior chamber of the eye following different treatments as compared to vehicle-treated control. Both mycophenolate 2% suspension (VT-1908) and mycophenolate mofetil ointment (VT-1910) reduced immune cells in anterior chamber. (**e**) Representative H&E staining showing various degrees of immune cell infiltration in anterior eye anatomies after BSA challenge in sensitized rabbits, and reduction in infiltration with different treatments. All images were captured at 4X magnification

We next tested the efficacy of the topical mycophenolate application using a standard model of uveitis [30]. Rabbits were first sensitized with BSA in complete Freund’s adjuvant emulsion, administered subcutaneously on days 1, 3, and 5. On day 9, we rechallenged the sensitized rabbits with an intravitreal eye injection of 50 µg BSA in 100 µL of sterile water. As shown in Fig. 5a–e, this resulted in a significant inflammatory response in the anterior chamber of the eye, characterized by increased flare and opacity and infiltration of immune cells. Consistent with clinical observations, the application of prednisone steroid suspension significantly reduced the uveitic score (flare and opacity) as well as decreased the infiltration of immune cells. Both mycophenolate mofetil 2% ointment and mycophenolate sodium 2% suspension eye drop resulted in a significant reduction in the uveitic score as well as infiltration of immune cells, with the latter being marginally superior. No statistical difference was seen between the steroid-treated and mycophenolate-treated groups.

**Table 2:**
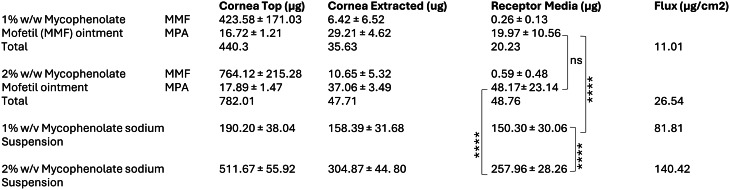
Mycophenolate penetration through cornea using a Franz assay. Total drug amount in each compartment was quantified 2 h post application of the formulations

Data shown are mean ± SD (*n* = 5–6, ****p < 0.0001, ANOVA followed by Tukey’s test. MPA: Mycophenolic acid)

## Discussion

Uveitis causes a high degree of visual morbidity, severely impacting patients’ quality of life [8, 31] and posing a significant burden on economic productivity and health care resource use [8, 32]. Classical treatment of anterior uveitis, the most common form of uveitis, relies on topical corticosteroids, which are associated with significant limiting side effects. Our study shows that topically applied mycophenolate can be as effective as steroid prednisone for the treatment of anterior uveitis. Indeed, mycophenolate (1–1.5 g BID oral) has been used clinically to treat patients with anterior uveitis responding poorly or refractory to corticosteroids, or those who develop severe IOP elevation in response to topical corticosteroids. The fact that we can achieve efficacy comparable to prednisone with topical mycophenolate at a maximal dose of 2 mg (100 µl of 2% formulation), i.e. a fraction of the 1–1.5 g oral mycophenolate dose that a patient is currently clinically administered, together with the published clinical data that mycophenolate can be used in conditions where steroids are contraindicated, offers a potential path to clinical translation of topical mycophenolate formulations for the treatment of uveitis.

Mycophenolic acid is a potent noncompetitive reversible inhibitor of IMPDH. It preferentially binds to human type II IMPDH with a K_i_ value of 6–10 nM or ~0.004335 µg/mL [26]. Our ocular pharmacokinetics study reveals that we can achieve significantly higher concentrations with topical application of mycophenolate than needed to inhibit IMPDH2. While the mycophenolate sodium suspension resulted in a faster and higher mycophenolate concentration in the anterior chamber, the ointment also resulted in intraocular concentrations higher than needed to inhibit IMPDH2, which could explain the overall similar efficacy with uveitic score in vivo despite significant quantity of drug being lost due to the inability to cross the corneal barrier.

Interestingly, while mycophenolate has been used clinically, and the structural basis of mycophenolic acid binding to IMPDH2 has been studied in different species, the binding of mycophenolate to human IMPDH2 had not been defined in detail. The model of human IMPDH2 in complex with the IMP and MPA shown in Fig. 2 extends our understanding of the binding or the IMP substrate and MPA inhibitor to the human enzyme. It allows a detailed comparison with the earlier, partial structure of the hamster enzyme [26] and may provide a foundation for the development of novel MPA derivatives with more favorable pharmacokinetic properties, for example through modifications of the phenolic oxygen responsible for MPA inactivation by glucuronidation [33]. The multiple conformations of the human IMPDH2 tetramer suggested by the present study are also of interest in view of recent kinetic, small angle X-ray and cryo-EM studies demonstrating the presence of higher order structure in response to different levels of adenine and guanine nucleotide binding to the regulatory domain [34–36] and highlighting the importance of end-product, allosteric regulation of IMPDH2 activity.

In conclusion, we have demonstrated the acute ocular safety and efficacy, underpinned by ocular pharmacokinetics, of topical mycophenolate formulations as a non-steroidal option for the treatment of anterior uveitis. Steroids are used widely at present to treat inflammation in the eye, but we have shown that topical application of mycophenolate is as effective and devoid of the complications associated with the use of steroids. Further development will need additional chronic repeat-dose safety studies, although the established safety data with systemic administration of higher doses of mycophenolate somewhat mitigates the risks. Although we achieved therapeutically effective concentrations in the target tissues, future work could focus in increasing the penetration through the corneal barrier. Topical application of mycophenolate merits clinical evaluation and may have broad application for treating local inflammatory disease beyond uveitis. Finally, the structural analysis of human IMPDH2 in complex with IMP and MPA presented here offers a strong foundation for further development of mycophenolate derivatives for clinical use.

## Electronic supplementary material

Below is the link to the electronic supplementary material.

Supplementary Material 1

## Data Availability

No datasets were generated or analysed during the current study.
