# Supplementary material for: Topical mycophenolate for the treatment of uveitis-associated inflammation
Source: J Ophthalmic Inflamm Infect. 2026 Feb 6;16:8. doi: 10.1186/s12348-026-00569-y (PMC12913868; doi:10.1186/s12348-026-00569-y)
Supplement: Supplementary file 1 — Supplementary Material 1 [file 12348_2026_569_MOESM1_ESM.pdf]

Supplementary Table.1.
Table 1: GRADING OF OCULAR LESIONS
Cornea
Opacity: degree of density (reading should be taken from most dense area)

No ulceration or opacity...	0
Scattered or diffuse areas of opacity (other than slight dulling of normal lusture); details of iris clearly visible	1
Easily discernible translucent areas; details of iris slightly obscured.........................	2
Nacrous area; no details of iris visible; size of pupil barely discernible......................	3
Opaque cornea; iris not discernible through the opacity......................................	4

Maximum possible score: 4

Area of cornea involved:

Zero.	0
One quarter (or less) but not zero	1
Greater than one quarter, but less than half	2
Greater than half, but less than three quarters	3
Greater than three quarters, up to whole area	4

Iris

Normal..	0
Markedly deepened rugae, congestion, swelling, moderate circumcorneal hyperaemia; or injection; iris reactive to light (a sluggish) reaction is considered to be an effect.........	1
Hemorrhage, gross destruction, or no reaction to light..	2

Maximum possible score : 2

Conjunctivae
Redness (refers to palpebral and bulbar conjunctivae; excluding cornea and iris)

Normal..	0
Some blood vessels hyperaemic (injected)..	1
Diffuse, crimson colour; individual vessels not easily discernible...........................	2
Diffuse beefy red..	3

Maximum possible score: 3

Chemosis
Swelling (refers to lids and / or nictating membranes)

Normal..	0
Some swelling above normal...	1
Obvious swelling, with partial eversion of lids..	2
Swelling, with lids about half closed...	3
Swelling, with lids more than half closed..	4

Maximum possible score : 4

Suppl. Table.2. Stability of mycophenolate in different formulations over time. Data is shown as mycophenolate concentration in each formulation as a % of the baseline (T0) mycophenolate concentration.

		**Ointment**		**Suspension eyedrop**	
		**1% w/w**	**2% w/w**	**1%w/v**	**2%w/v**
Initial Assay		100.39	99.88	97.84	100.85
1 Month	25° C./60% RH	96.41	96.21	97.04	103.02
	40° C./75% RH	111.68	101.85	96.16	102.14
2 Month	25° C./60% RH	98.88	99.87	97.12	100.98
	40° C./75% RH	108.70	100.22	94.40	99.55
3 Month	25° C./60% RH	99.01	98.91	95.12	97.57
	40° C./75% RH	116.84	112.54	93.15	97.16
6 Month	25° C./60% RH	103.08	99.91	93.59	94.40
	40° C./75% RH	102.35	109.12	91.44	95.51

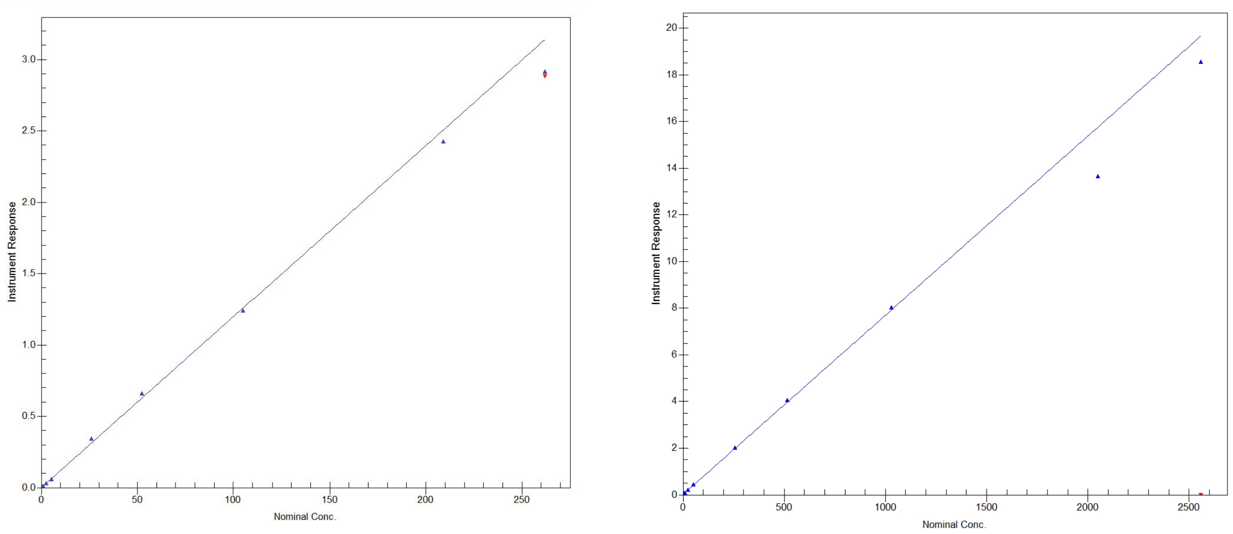

Suppl. Figure. 1. Calibration standard curves for (a) mycophenolate mofetil (ng/ml). Regression Method = LINEAR - Weighting Factor = 1/X"2 Response = Slope • Cone + Intercept; Slope = 0.01 19887 Intercept = -0.000505212 R-Squared = 0.9954. **(b)** mycophenolic acid (ng/ml). Regression Method = LINEAR - Weighting Factor = 1/X”2 Response = Slope • Cone + Intercept; Slope = 0.00768044 Intercept = 0 0175293, R-Squared = 0.9942.
